# Cyclophosphamide induces ovarian granulosa cell ferroptosis via a mechanism associated with HO-1 and ROS-mediated mitochondrial dysfunction

**DOI:** 10.1186/s13048-024-01434-z

**Published:** 2024-05-18

**Authors:** Hui Chen, Ping Nie, Jingling Li, Yongqi Wu, Bo Yao, Yabing Yang, Gendie E. Lash, Ping Li

**Affiliations:** 1 Department of Pathology, Jinan University School of Medicine, Guangzhou, 510632 China; 2 Guangdong Second Provincial General Hospital, Postdoctoral Research Station of Basic Medicine, Jinan University School of Medicine, Guangzhou, 510317 China; 3 Guangzhou Institute of Pediatrics, Guangzhou Women and Children’s Medical Center, Guangzhou Medical University, Guangzhou, 510623 China

**Keywords:** Granulosa cell, Ferroptosis, Cyclophosphamide, Heme oxygenase 1, Mitochondria

## Abstract

Abnormal granulosa cell (GC) death contributes to cyclophosphamide (CTX) induced primary ovarian insufficiency (POI). To investigate the contribution of GCs to POI, gene profiles of GCs exposed to CTX were assessed using RNA-Seq and bioinformatics analysis. The results showed the differentially expressed genes (DEGs) were enriched in the ferroptosis-related pathway, which is correlated with upregulated heme oxygenase 1 (HO-1) and downregulated glutathione peroxidase-4 (GPX4). Using CTX-induced cell culture (COV434 and KGN cells), the levels of iron, reactive oxygen species (ROS), lipid peroxide, mitochondrial superoxide, mitochondrial morphology and mitochondrial membrane potential (MMP) were detected by DCFDA, MitoSOX, C11-BODIPY, MitoTracker, Nonylacridine Orange (NAO), JC-1 and transmission electron microscopy respectively. The results showed iron overload and disrupted ROS, including cytoROS, mtROS and lipROS homeostasis, were associated with upregulation of HO-1 and could induce ferroptosis via mitochondrial dysfunction in CTX-induced GCs. Moreover, HO-1 inhibition could suppress ferroptosis induced GPX4 depletion. This implies a role for ROS in CTX-induced ferroptosis and highlights the effect of HO-1 modulators in improving CTX-induced ovarian damage, which may provide a theoretical basis for preventing or restoring GC and ovarian function in patients with POI.

**Supplementary Information:**

The online version contains supplementary material available at 10.1186/s13048-024-01434-z.

## Introduction

Cyclophosphamide (CTX) is a widely used chemotherapeutic agent and effectively improves the survival of various cancer patients [1]. However, CTX is an alkylating agent and thus is known as a cell cycle independent drug, and thus associated adverse effects in cancer patients have been linked to oxidative stress generation in normal tissues [2]. In the ovary CTX may destroy the follicular pool, leading to primary ovarian insufficiency (POI). The main mechanism of POI caused by CTX may involve targeting oocytes directly or inducing oocyte death indirectly by injuring granulosa cells (GCs) [3]. GCs play an essential role in determining the fate of the follicle and abnormal follicle development is the main phenotype of POI. Despite extensively reported GCs damage in developing follicles exposed to CTX, the nature of these mechanisms in GCs which result in depletion of ovarian reserve, sometimes termed ‘burnout’ theory is still under debate and this hampers the further development of therapeutic interventions [3, 4].

Ferroptosis is an iron-, lipid- and oxidative stress-dependent form of non-apoptotic cell death. Severe oxidative stress can cause cell death and GCs are vulnerable to reactive oxygen species (ROS) attack. CTX can be converted to its active metabolite 4-hydroxycyclophosphamide (4HC) and produce more ROS [2]. There is strong evidence that ROS are involved in the initiation of cell death in GC upon exposure to CTX [5, 6]. Numerous studies have shown that POI occurs with the apoptosis of GCs, and recent work suggests ferroptosis of GCs contributes to cisplatin (CIS)-induced POI in vivo and in vitro [6, 7]. Generally, mitochondria are particularly prone to oxidative damage, which are thought to contribute significantly to ovarian pathology [8]. However, it is considered that the mitochondria are dispensable for ferroptosis [9]. It is unclear whether the relationship exists, and whether ROS-mediated mitochondrial dysfunction lead to GC ferroptosis in CTX-induced POI.

Studies have shown that genetic factors are associated with 7% of POI case [10]. Heme oxygenase 1 (HO-1), an enzyme that degrades heme to ferrous iron, contributes to ferroptosis in several disease settings. HO-1 is mainly expressed in GCs in ovaries and HO-1-deficient mice showed deficient ovulation [11]. In autoimmune POI mice, HO-1-mediated immunodeficiency has been found to improve ovarian reserve capacity through the HO-1 pathway [12]. This evidence revealed HO-1 counteracts immune-mediated inflammation mechanism in POI. It remains unclear whether HO-1 participates in the ferroptosis of GC in CTX-induced POI.

Granulosa cell lines treated with 4HC, the pharmacologically active moiety of CTX, have been widely used for in vitro studies involving POI [13]. In this study, the transcriptome response in 4HC-treated COV434 (human immortalized GC cell line) was analyzed with RNA-Sequencing (RNA-Seq). We revealed that CTX trigged ferroptosis in COV434 and KGN cells, and its regulation by HO-1 that might predispose GCs to exhibit mitochondrial dysfunction and thereby be involved in CTX- induced POI.

## Materials and methods

### Cell culture and treatment

The human GC cell lines COV434 and KGN cell were purchased from the Cell Bank, Procell Life Science Technology Co., Ltd (Wuhan, China). COV434 cells were cultured in DMEM medium (Gibco, Massachusetts, USA), and KGN cell were cultured in DMEM/F12 (Gibco, Massachusetts, USA) with 10% fetal bovine serum (FBS) (5% CO_2_, 20% O_2_, 37 °C). The effects of 4HC (Santa Cruz Biotechnology, TA, USA) on COV434 or KGN cells were evaluated at various concentrations (from 0 to 100 µM) and different times (from 12 to 48 h). The OD value was detected by the CCK-8 method (Beyotime Biotech, Nanjing, China); the optimal concentration for subsequent experiments was used. Specific inhibitor ferrostatin-1 (Fer-1, MedChemExpress, New Jersey, USA) was added 20 µM for 1 h before 4HC treatment [14].

The siRNAs against human HO-1 (siHO-1) and negative control (siNC) were purchased from Guangzhou Ribobo Co., Ltd (Gunagzhou, China). The sequences are presented in Table S1. Cells were transfected with siRNA using lipofectamine as previously described [15].

### Cell viability assay

Cell viability was determined using a CCK-8 kit (Beyotime Biotech, Nanjing, China) as previously described [16]. Equal amounts of cells were seeded in 96-well plates and incubated with 4HC at different concentrations for various times (from 0 to 48 h). Optical density was measured at a wavelength of 450 nm using a microplate reader (SpectroAma™ 250, Winooski, USA).

### Iron assay

Intracellular iron content was measured by Iron Colorimetric Assay Kit (Abcam, Cambridge, UK). To measure total iron, 5 µL Iron Reducer was added to each sample well to reduce Fe^3+^ to Fe^2+^, and incubated at 37 °C for 30 min. 100 µL Iron Probe was added to each well containing the Iron Standard and/or test samples. Total iron (Fe^2+^ + Fe^3+^) concentrations can be determined from the standard curve. The iron concentration was expressed as ng/µL.

### ROS levels assay

Intracellular ROS (cytoROS) was evaluated by a 7-dichlorodihydrofluorescein diacetate fluorescent probe (DCFDA, Beyotime Biotech, Nanjing, China) [17]. 2 × 10^5^ cells were stained with DCFDA solution dissolved at 50 mM in pre-warmed PBS for 30 min at 37 ºC and measured at a 488 nm excitation wavelength and 525 nm emission wavelength (BioTek Instruments, Winooski, VT, USA).

Mitochondrial ROS (mitoROS) production was quantified by MitoSOX™ Red (Thermo Fisher Scientifific, MA, USA). The cells were loaded in serum- free medium with 2.5 µM MitoSOX Red for 30 min in the dark. The fluorescence was monitored with a microplate reader set using a 396 nm excitation wavelength and 610 nm emission wavelength.

The activity of malondialdehyde (MDA) and glutathione (GSH) in the cells were determined using commercial assay kits (Nanjing Jiancheng, Nanjing, China) as previously described [18]. The ROS levels are presented as the percentage relative to the value in the control group.

### Lipid peroxidation assay

Lipid peroxides were detected by the C11-BODIPY^581/591^ probe in live cells (Thermo Fisher Scientifific, MA, USA). The cells were seeded in 6-well plates and loaded with 5 µM C11-BODIPY^581/591^ for 30 min then washed three times with PBS. The spectrofluorometric analysis was performed recording the fluorescence intensity spectra at 500–650 nm emission wavelengths. The lipid peroxidation value was determined as a ratio between the fluorescence emission peak value at ∼ 520 nm and the sum of the fluorescence emission peak values at ∼ 520 and ∼ 590 nm. Data were collected from at least 20 000 cells. Each experiment was performed in triplicate.

The green fluorescence intensity was observed under a fluorescent inverted microscope and was calculated by Image J software. After oxidation, the probe shifts the excitation and emission wavelengths to 488/510 nm.

### Mitochondrial assays

Mitochondrial mass was measured by staining live cells with MitoTracker Red CMXRos (Thermo Fisher Scientifific, MA, USA) and Nonyl Acridine Orange (NAO) (Invitrogen, Carlsbad, CA). The nuclei were labeled with 4’, 6-diamidino − 2-phenylindole (DAPI, Beyotime, Shanghai, China). Mitochondrial fluorescence intensity was observed and photographed by fluorescence microscopy (Echo Laboratories, New York, USA).

Mitochondrial membrane potential (MMP) was estimated by JC-1 (Beyotime, Nanjing, China). Cells were incubated with 5 µM JC-1 for 30 min in the dark then washed and resuspended. The JC-1 aggregates or monomers were detected by immunofluorescence microscopy. JC-1 selectively enters into mitochondria where it can exist in two forms, monomeric or aggregate, depending upon the MMP. The green-shifted monomers tend to predominate under conditions with low MMP, whereas the red-shifted aggregates are favored under conditions with high MMP [19].

### RNA sequencing and bioinformatics analysis

Total RNA was isolated from cell samples using TRIzol (Thermo Fisher Scientific, MA, USA). RNA sequencing was performed by MGI Technology Co., Ltd (BGI, Shenzhen, China). Differentially expressed genes (DEGs) between different samples were detected as those with a fold change ≥ 2 and a *p* value < 0.05 using the R package DESeq 2 (version: 1.34.0), and the DEGs were further analyzed by the GO (gene ontology) and KEGG (Kyoto Encyclopedia of Genes and Genomes) databases to assess their functional enrichment. The RNA-seq data of control and 4HC-treated COV434 cells were submitted to the Gene Expression Omnibius (GSE243720) ( https://www.ncbi.nlm.nih.gov/geo/query/acc.cgi?acc=GSE243720).

### Data analysis

GO and KEGG pathway enrichment analysis for DEGs were performed using the “org.Hs.eg.db” package (version:3.15.0) and “clusterProfiler” (version:4.4.4) of R. GSEA 4.2.3 (gene set enrichment analysis software) was performed to identify whether a set of genes associated with specific GO terms or pathways showed significant differences between the two groups. Enrichment scores and *p*-values were calculated with default parameters.

The STRING database (https://string-db.org/) was downloaded and used to filter protein-protein interaction (PPI) among DEGs. For screening ferroptosis-related DEGs, collection and collation of ferroptosis-related genes from the “FerrDb” database was performed [20]. Identification of hub genes was based on the PPI network construction. Through importing the overlapping genes into the Search Tool for the Retrieval of Interacting Genes database [21], the interaction relationships among the proteins encoded by the overlapping genes were searched. Results were visualized as the PPI network by Cytoscape software. In a co-expression network, Maximal Clique Centrality [22] algorithm was used to select the hub genes. The MCC score of each node was calculated by the Cytohubba plugin of Cytoscape.

### RT-qPCR and western blotting analysis

RT-qPCR and western blot were performed as previously described [15]. The specific primers and antibodies are presented in Table S1 and S2. The expression of genes was normalized to β-actin or GAPDH. The results are representative of three independent experiments.

### Transmission electron microscopy

Cells (1 × 10^6^) were prefixed with glutaraldehyde for 2 h (Macklin, Wuhan, China) and embedded in osmium acid solution. Subsequent processing was completed as previously described [18]. The ultrastructure of the samples was observed at the Analysis and Testing Center of Jinan University.

### Statistical analysis

GraphPad Prism 9 Software (GraphPad Software, CA, USA) was used for all analyses. Comparisons between groups were performed by unpaired t-test, and data with unequal variance were compared with the Mann-Whitney U-test. ANOVA was used for multiple comparisons of data from more than two groups. Results are presented as means ± SEM (*n* = 3), and *p* < 0.05 was considered statistically significant.

## Results

### Induction of ferroptosis in CTX-treated GCs

**Fig. 1:**
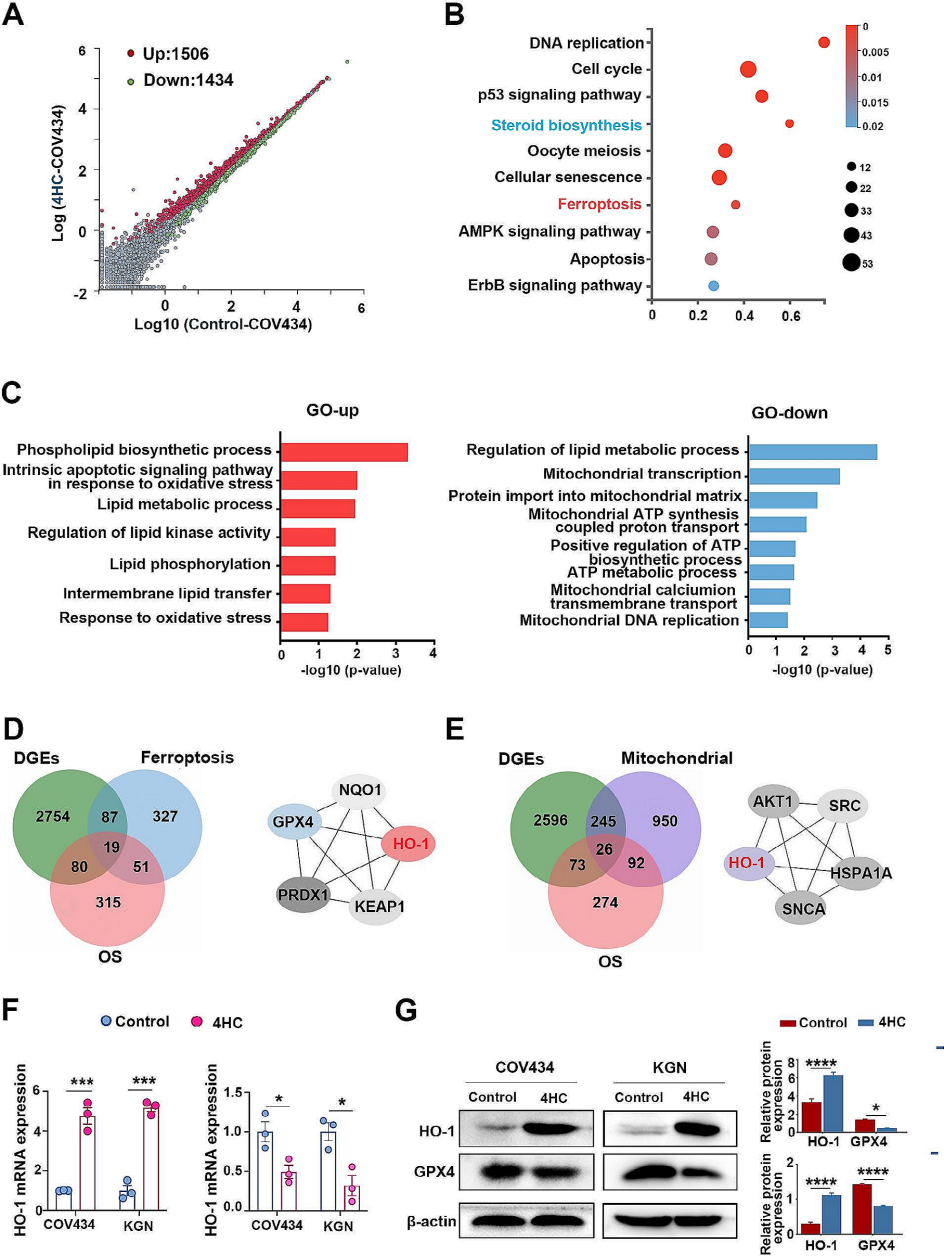
The ferroptosis pathway was involved in 4HC-treated GCs. **(A)** Scatter plot (red, upregulated; green, downregulated), **(B)** KEGG enrichment analysis and **(C)** GO enrichment items for differentially expressed genes (DEGs) of RNA-seq in COV434 cells in response to 4HC treatment compared with control. **(D)** Venn plots and STRING analysis the gene intersections taken between the DEGs, oxidative stress- and ferroptosis- gene sets. **(E)** Venn plots and STRING analysis the gene intersections taken between the DEGs, oxidative stress- and mitochondrial- gene set. **(F)** Relative expression of the HO-1 and GPX4 mRNAs and (G) proteins in COV434 and KGN cells treated with 4HC (15 µM and 25 μm, respectively) for 24 h. Data are presented as the means ± SEM from more than three independent experiments; *p < 0.05, ***p < 0.001 and ****p < 0.001 for the indicated comparisons

To investigate the biological process underlying the ovarian toxicity effects of CTX, bulk RNA sequencing was used to examine DEGs in COV434 cells treated with 15 µM 4HC. In total, 1506 DEGs (red) were upregulated and 1434 DEGs (green) were downregulated (Fig. 1A). Functional enrichment analysis showed the gene cluster enrichment of categories, ferroptosis and cellular senescence were upregulated, and steroid biosynthesis was down-regulated (Fig. 1B). Enrichment GO analysis showed the downregulated genes were enriched in the terms mitochondrial ATP synthesis, oxidative phosphorylation and re-sponse to oxidative stress, and the upregulated genes were enriched in hormone and oocyte development (Fig. 1C). Then, the DEGs, the ferroptosis-related genes, and oxidative stress (OS)-related genes were overlapped, and a total of 19 genes intersected and a PPI network was constructed using STRING online database and Cytoscape (Fig. 1D and Supplementary Figure S1A). The DEGs, the mitochondial-related genes, and OS-related genes were overlapped, and a total 26 genes intersected and a PPI network was constructed (Fig. 1E and Figure S1B). Based on the maximal clique centrality (MCC) score, the top highest genes were finally identified as hub genes and the gene symbols, full names and MCC scores are displayed in Table S1. Finally, the ferroptosis-related (GPX4, NQO1, HO-1, PRDX1, KEAP1) or OS-related DEGs (AKT1, SRC, HO-1, SNCA, HSPA1A) were analyzed using CytoHubba, respectively, and HO-1 was obtained in both sets of hub genes (Fig. 1D and E). Accordingly, in 4HC treated COV434 and KGN cells 15 µM and 25 µM for 24 h, respectively, the expression of HO-1 was significantly unregulated and GPX4 was downregulated compared to the control cells by qRT-PCR (Fig. 1F) and Western blot analysis (Fig. 1G).

### CTX triggers iron overload, increased ROS and lipid peroxidation in GCs

**Fig. 2:**
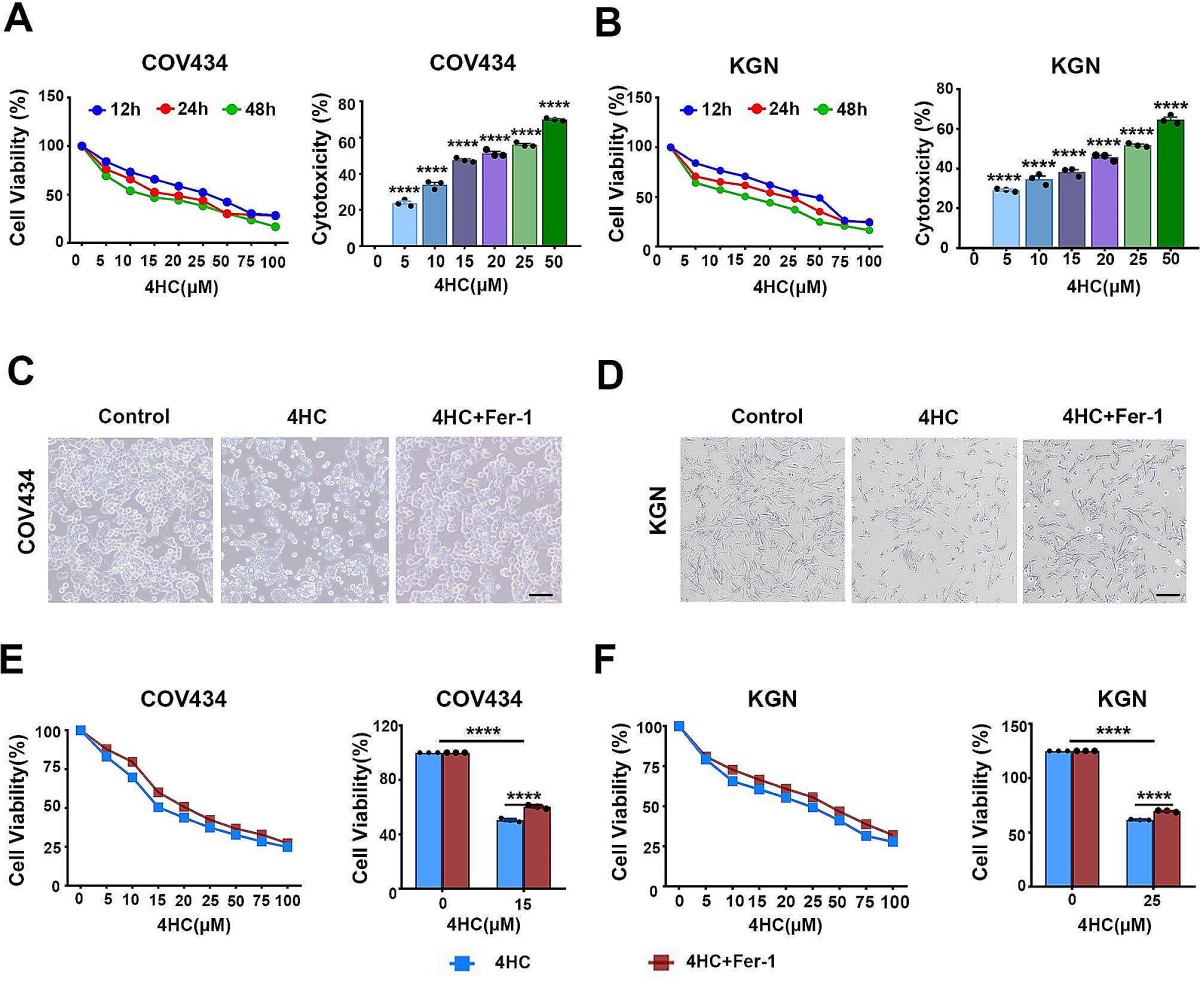
Ferroptosis inhibitor reversed 4HC-induced cytotoxicity. **(A)** Dose- and time-response curve used to estimate IC50 values of 4HC in COV434 and **(B)** KGN cells, accompanied by increased toxicity. **(C)** Morphological changes of COV434 and **(D)** KGN cells treated with 4HC with or without Fer-1 for 24 h. **(E)** Dose-response curve of 4HC was preincubated with or without Fer-1 (20 µM) for 24 h in COV434 and **(F)** KGN cells. Cell viability and toxicity was expressed as percentage with respect to control and 4HC at different concentrations. Each point represents the mean of quadruplicates. Scale bars = 200 μm. ****p < 0.0001 for the indicated comparisons

To confirm whether ferroptosis contributed to CTX-induced ovarian toxicity and the underlying mechanisms related to cellular iron release, the inhibitor of ferroptosis (Fer-1) was used in vitro. The viability of the COV434 and KGN cell lines treated with 4HC (0-100 µM) for 12, 24–48 h was significantly reduced in a dose- and time-dependent manner compared with that of the control groups, accompanied by increased cytotoxicity. The IC50 values of 15 µM in COV434 and 25 µM in KGN at 24 h were used in subsequent experiments (Fig. 2A and B). 4HC treatment also altered the cell morphology under an inverted microscope, which was characterized by a reduced cell size with wrinkled cell membranes and broadened intercellular gaps in COV434 and KGN cells (Fig. 2C and D). Notably, 4HC-induced cell death and morphological changes were significantly attenuated in the presence of Fer-1 (Fig. 2C-F).

**Fig. 3:**
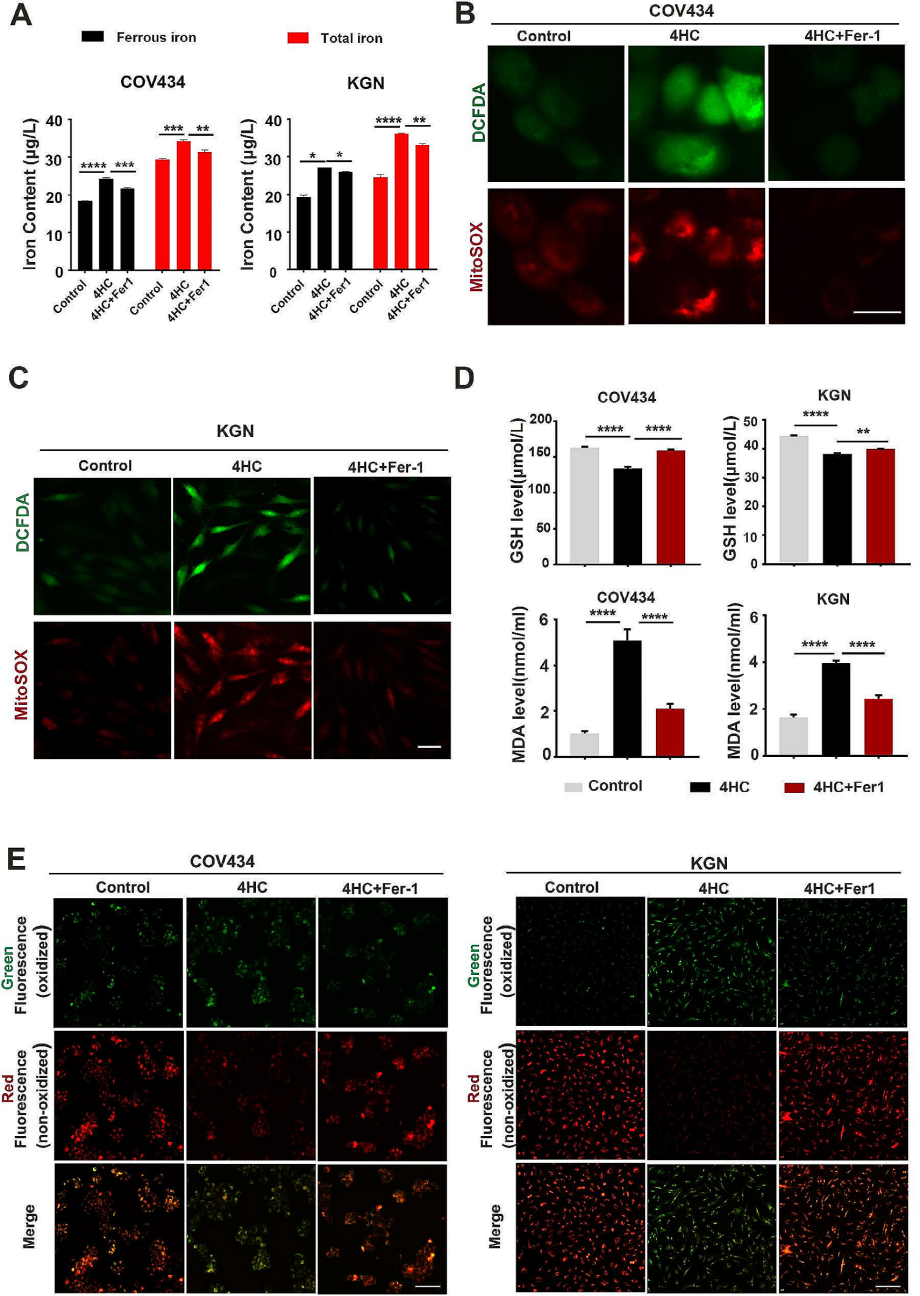
Ferroptosis inhibitor blocked 4HC-induced iron overload, oxidative stress and lipid peroxidation. **(A)** Intracellular iron content was assessed by the colorimetric ferrozine-based assay in control and cells treated with CTX with or without Fer-1. **(B)** Cyto- and mito-ROS levels were measured using the DCFDA (green) and MitoSOX fluorescence probe (red) method in COV434 and **(C)** KGN cells, respectively. **(D)** The level of GSH and MDA assay in control and cells treated with CTX with or without Fer-1. **(E)** Cells stained with peroxidation probe (C11-BODIPY581/591) in control and cells treated with CTX with or without Fer-1. Scale bars = 50 μm in B and C, and 100 μm in D. **p < 0.01 and ****p < 0.0001 for the indicated comparisons

Since ferroptotic cell death involves the accumulation of intracellular iron, resulting in the production of cytosolic and lipid ROS, the level of intracellular iron content was measured. In COV434 cells, Fe^2+^ and total iron were found to be enhanced (18.35 ± 0.09 vs. 24.21 ± 0.36, *P* < 0.0001; 29.36 ± 0.23 vs. 34.24 ± 0.38, *P* = 0.0005) after 4HC treatment compared to controls. In KGN cells, Fe^2+^ and total iron were found to be enhanced (19.31 ± 0.58 vs. 27.03 ± 0.02, *P* = 0.0107; 24.49 ± 0.66 vs. 36.19 ± 0.08, *P* < 0.0001;) after 4HC treatment compared to controls. In contrast, Fe^2+^ and total iron were found to be reduced in COV434 (24.21 ± 0.36 vs. 21.63 ± 0.18, *P* = 0.0007; 34.24 ± 0.38 vs. 31.35 ± 0.61, *P* = 0.008) and KGN cells (27.03 ± 0.02 vs. 25.99 ± 0.11, *P* = 0.0141; 36.19 ± 0.08 vs. 33.06 ± 0.43, *P* = 0.0068;) after Fer-1 treatment compared to the 4HC group (Fig. 3A), suggesting that Fer-1 could reverse the CTX-induced increase in intracellur iron content. Furthermore, the cytoROS and mtROS state was assessed by staining with DCFDA and MitoSOX. Cell DCFDA is a widely used total ROS indicator which can be oxidized by ROS, the resulting dichlorofluorescein (DCF) exhibits high fluorescence [23]. MitoSOX™ Red is specifically targeted to mitochondria and reports the intensity of generation of superoxide radicals within mitochondria [24]. Intracellular and mitochondrial ROS formation was increased during 4HC induction and those parameters were significantly reduced after the addition of Fer-1 to the culture in both COV434 and KGN cells (Fig. 3B and C).

Glutathione (GSH) exhaustion is a critical event during ferroptosis-mediated cell death [25]. When 4HC was administered, the level of GSH was decreased in COV434 (163.4 ± 1.06 vs. 134.25 ± 2.11, *P* < 0.0001) and in KGN cells (44.47 ± 0.11 vs. 38.14 ± 0.36, *P* < 0.0001) (Fig. 3D). MDA is a lipid peroxidation byproduct [26], lipid peroxides were aggravated in 4HC treated COV434 (1.02 ± 0.1 vs. 5.08 ± 0.49, *P* < 0.0001) and KGN cells (1.65 ± 0.12 vs. 3.96 ± 0.09, *P* < 0.0001), as indicated by elevated MDA (Fig. 3D). C11-BODIPY^581/591^ staining results showed that oxidized C11-BODIPY^581/591^ was obviously detectable on the plasma membrane in COV434 and KGN cells after CTX treatment, suggesting lipid peroxides were accumulated preferentially in the plasma membrane of cells exposed to CTX. However, after Fer-1 treatment, GSH levels were increased and MDA levels were reduced (Fig. 3D), and the oxidized C11-BODIPY signal was prevented in both COV434 and KGN cells (Fig. 3E). These results suggest that 4HC significantly disrupts GCs antioxidant capacity and is responsible for lipid peroxidation accumulation. Fer-1 abrogates the effect caused by 4HC in both COV434 and KGN cells.

### CTX induced mitochondrial dysfunction in GCs

**Fig. 4:**
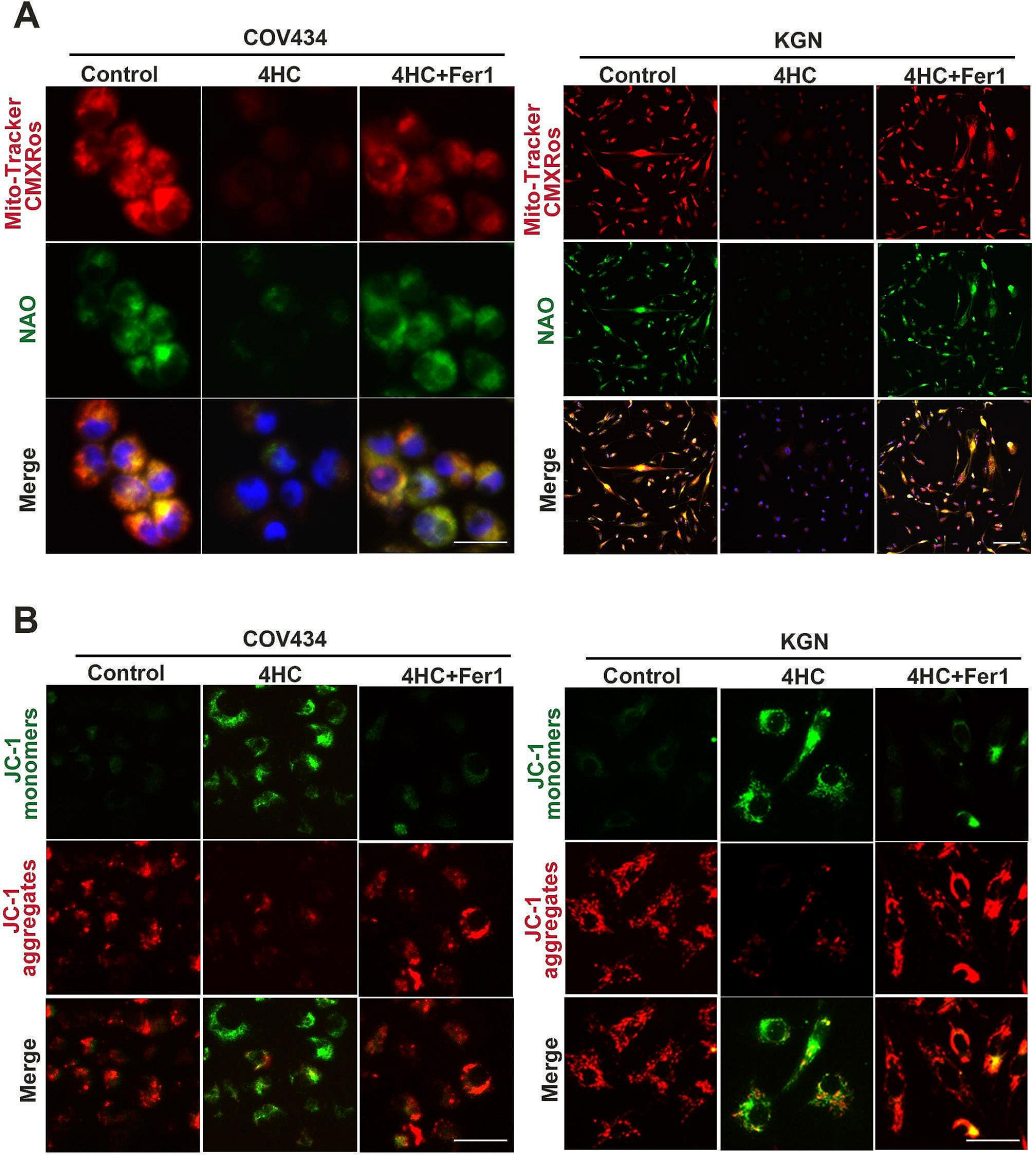
Ferroptosis inhibitor reversed mitochondrial dysfunction caused by 4HC. **(A)** Mitochondrial activity was assessed by Mitotracker (red) and NAO (green) staining; **(B)** cell mitochondrial membrane potential was assessed by JC-1 staining in control and cells treated with CTX with or without Fer-1 (red for aggregate, green for monomer). Scale bars = 100 μm in A and 25 μm in B

To characterize the mitochondrial metabolic state, the MMP sensitive Mito-Tracker Red and NAO were used as markers for mitochondrial mass [27]. The results showed that decreased red and green fluorescence intensity in 4HC treated-cells, suggesting 4HC inhibited mitochondrial activity and mass in GCs, and Fer-1 can rescue mitochondrial damage (Fig. 4A). Using JC-1 staining, the red or green fluorescence showed aggregates and monomers, reflecting high or low MMP, respectively. It showed MMP was reduced with 4HC induction, as evidenced by a pronounced decrease in JC-1 aggregate formation (with specific red fluorescence), and more free green puncta indicating JC-1 monomers, which was restored after treatment with Fer-1(Fig. 4B). The TEM results showed that compared with the control group, the cell membranes of COV434 cells in the CTX group were ruptured and blebbed, mitochondria atrophied and became smaller, mitochondrial ridges were reduced or even disappeared, and apoptotic bodies appeared. Compared with the CTX group, the COV434 cells a in the Fer-1 group were significantly improved (Fig. 7C).

### The effect of HO-1 on ferroptosis in CTX- induced GCs

**Fig. 5:**
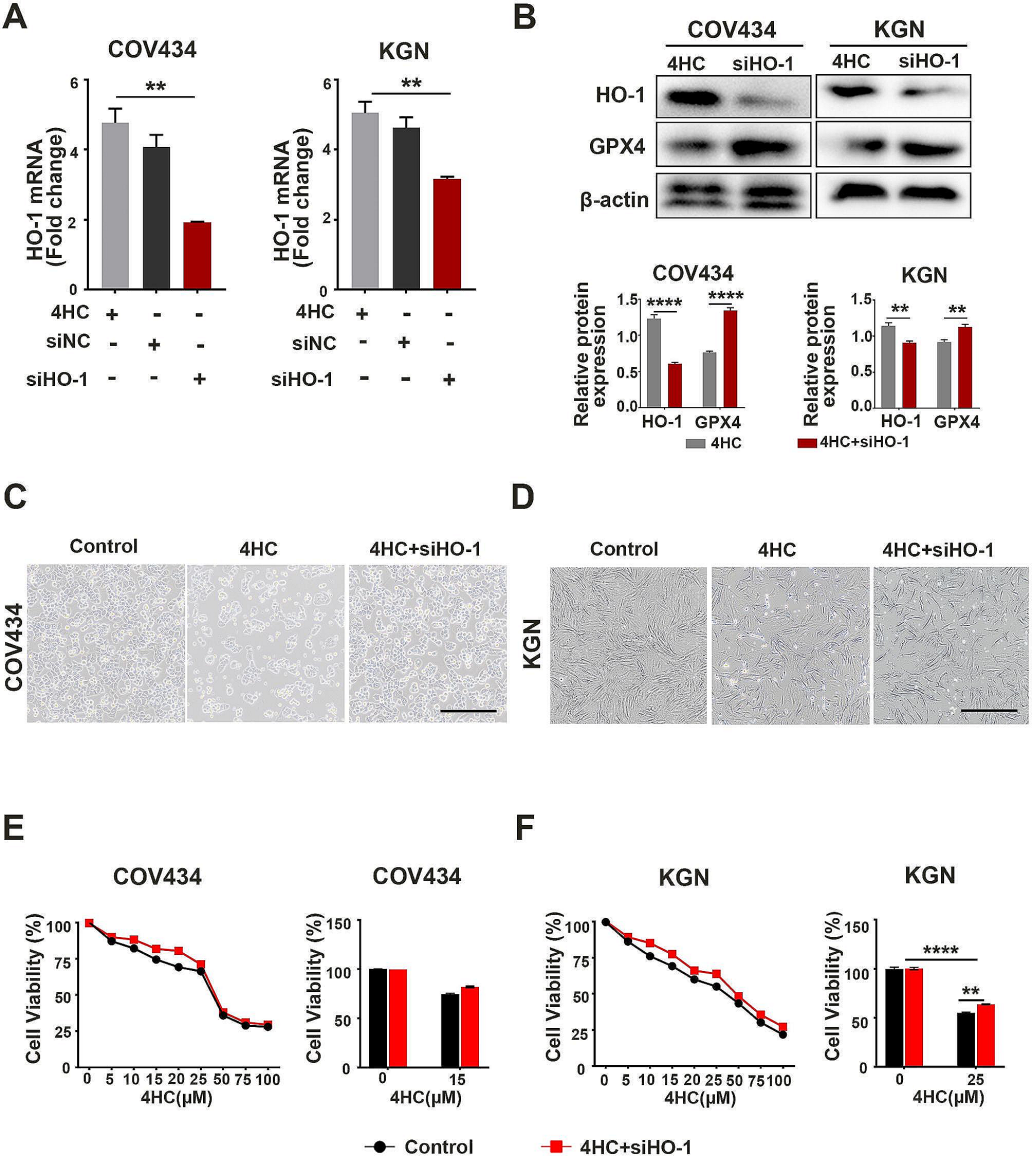
HO-1 Knockdown reduced the effect of CTX in GCs. **(A)** qPCR and **(B)** western blot analysis were used to measure the transfection efficiency and GPX4 expression of si-HO-1 in COV434 and KGN cells. **(C)** Morphological changes and **(D)** cell viability were examined in COV434 and KGN cells after HO-1 knockdown. Scale bars = 100 μm. **p < 0.01 and ****p < 0.0001 for the indicated comparisons

To confirm the activity of HO-1 contributes to GC ferroptosis and the underlying mechanisms in CTX-induced POI, HO-1 was knockeddown by siRNA and used in CTX-induced COV434 and KGN cells. qRT-PCR analysis showed that HO-1 mRNA content was reduced by 44% and 57% in COV434 and KGN cells, respectively (Fig. 5A). Accordingly, the western blot result showed that HO-1 protein was decreased in siRNA transfected cells compared with control cells (Fig. 5B). After CTX treatment with siRNA transfection, COV434 and KGN cells exhibited the restored morphological phenotype and cell viability, accompanied with the typical morphology for CTX-untreated cells (Fig. 5C-F).

**Fig. 6:**
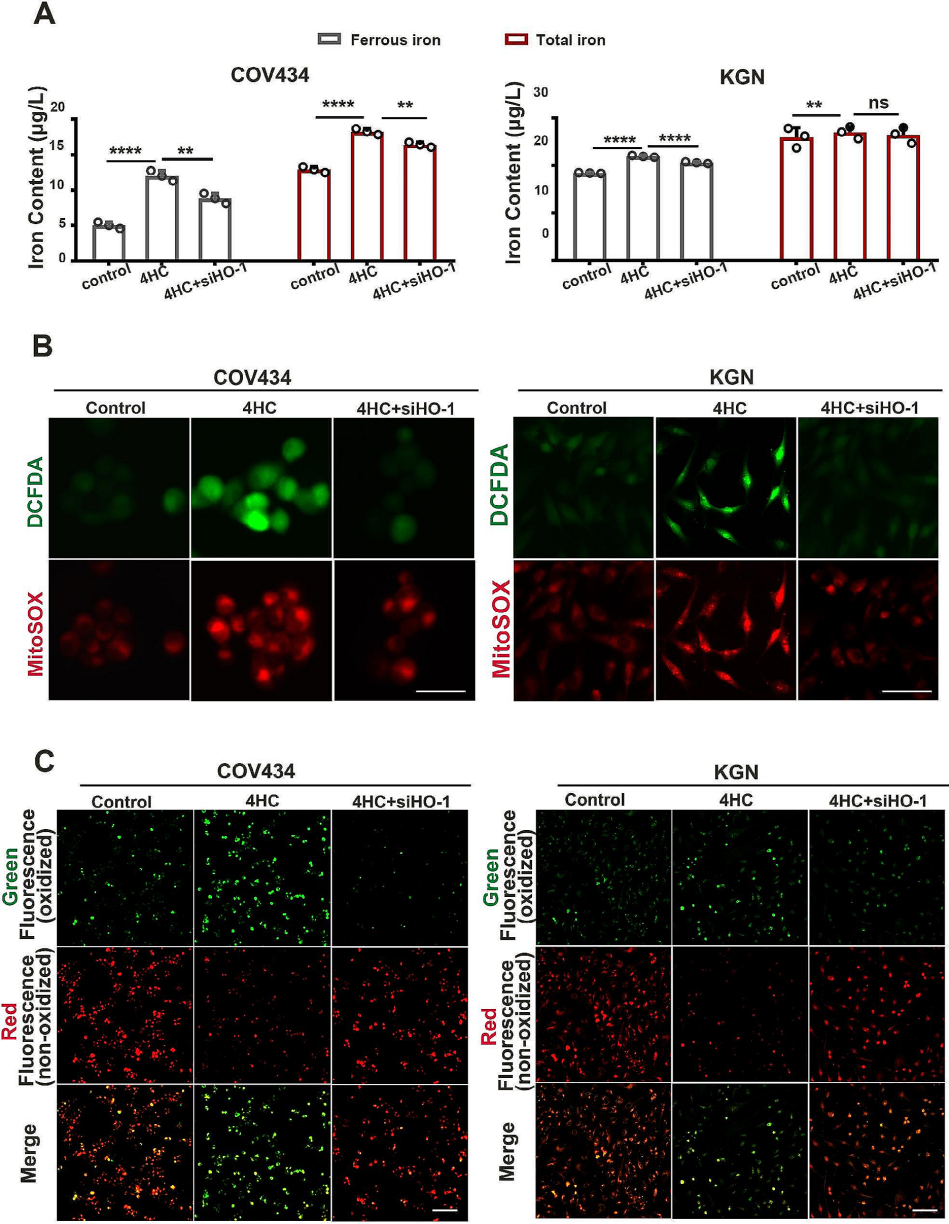
HO-1 Knockdown relieved iron overload and lipid peroxidation caused by 4HC. **(A)** Intracellular iron content was assessed by the colorimetric ferrozine-based assay in control and cells treated with CTX with or without siHO-1. **(B)** Cyto- and mito-ROS level was measured using the DCFDA (green) and MitoSOX fluorescence probe method (red) in COV434 and KGN cells, respectively. **(C)** Cells stained with peroxidation probe (C11-BODIPY581/591) in control and cells treated with CTX with or without siHO-1. Scale bars = 25 μm in B and 100 μm in C. **p < 0.01, and ****p < 0.0001 for the indicated comparisons

Further, in both COV434 and KGN cells, iron assay results showed that HO-1 knockdown reversed the CTX induced increase in intracellur iron levels. In COV434 cells, Fe^2+^ and total iron were found to be reduced (12.03 ± 0.42 vs. 8.84 ± 0.41, *P* = 0.0021; 18.27 ± 0.23 vs. 16.44 ± 0.2, *P* = 0.0028) in the siHO-1 group compared to the 4HC group. In KGN cells, Fe^2+^ was significantly reduced (21.95 ± 0.1 vs. 20.59 ± 0.1, *P* < 0.0001) while there was no significant difference in total iron content (27.99 ± 0.1 vs. 26.64 ± 0.24, *P* = 0.08) in the siHO-1 group compared to the 4HC group (Fig. 6A). DCFDA and MitoSOX immunofluorescence staining results showed that HO-1 knockdown reversed the CTX induced increases in cytoROS and mtROS formation (Fig. 6B). C11-BODIPY^581/591^ staining results showed that HO-1 knockdown prevented the oxidized fluorescence signal caused by 4HC (Fig. 6C).

**Fig. 7:**
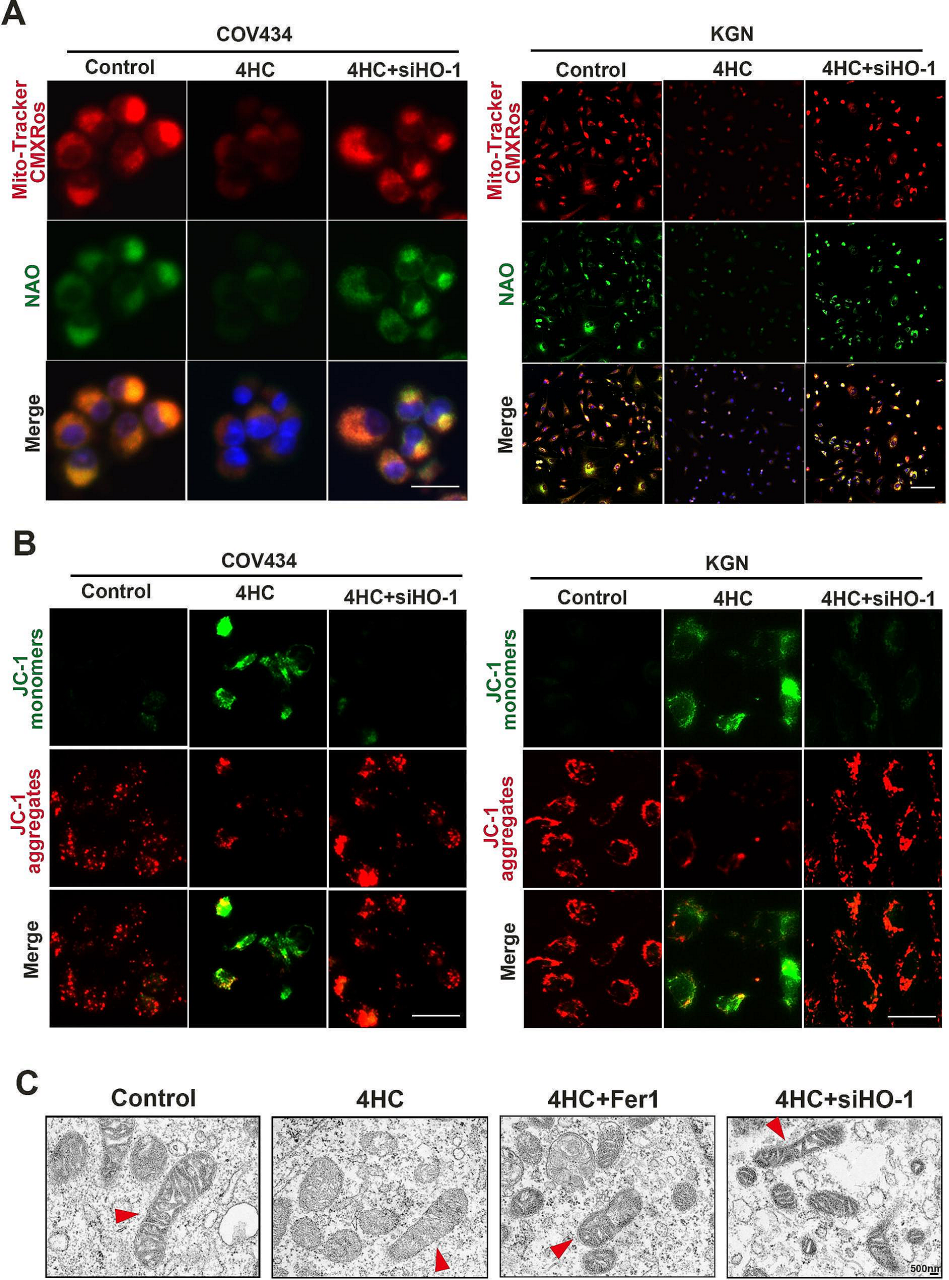
HO-1 Knockdown relieved mitochondrial dysfunction caused by 4HC. **(A)** Mitochondrial activity was assessed by Mitotracker (red) and NAO (green) staining; (**B** and **C**) cell mitochondrial membrane potential was assessed by JC-1 and TEM in control and cells treated with CTX with or without HO-1 knockdown. Scale bars = 100 μm in A and 25 μm in B

Moreover, HO-1 knockdown relieved CTX-induced mitochondrial activity and mass, as determined by the Mito-Tracker Red and NAO fluorescence assays (Fig. 7A). HO-1 knockdown also reduced the CTX-induced decreased monomer formation, reflecting a relative high MMP (Fig. 7B). Consistent with these data, TEM results results showed HO-1 knockdown improved the mitochondrial morphology and structure compared with CTX-treated cells (Fig. 7C).

## Discussion

Preventing GC death has been suggested to be a potential of strategy for fertility preservation [5, 6]. However, it is still debated by the field as to which regulated cell death pathway exists in GCs under oxidative stress, or how this pathway occurs in the lead up to POI [6]. Here, we demonstrated ferroptosis of GCs in ovarian damage induced by CTX. In CTX-treated GCs, HO-1 is upregulated and mediates oxidative stress, iron release and mitochondrial dysfunction. Inhibiting either ferroptosis or knockdown of HO-1 significantly relieved CTX-induced mitochondrial dysfunction, which demonstrated a potential therapeutic approach for treating and/or preventing CTX-induced POI, additionally enhancing our understanding of the pathogenesis of CTX-induced POI.

Fertility preservation is an important aspect of oncological care and chemotherapy-induced ovarian toxicity is a major challenge in oncofertility. Notably, the ferroptosis pathway is enriched and the related genes are expressed by the GC and oocyte during primordial follicle assembly, and this reflects the role of ferroptosis in follicle loss and POI [10, 28]. However, rather than initiating primary follicular depletion, GC may mainly act on follicle growth, acting as “burnout” [3]. That is, the injury to growing reduces its inhibitory effects on primordial follicles, thus resulting in activation of the primordial follicles. Although GC apoptosis is the major form of regulated cell death, recent studies have demonstrated that increased ROS triggered apoptotic and non-apoptotic (necroptosis) in GCs, which are responsible for atresia of primordial follicles have also been reported to be implicated in POI [6, 21, 29]. Several studies suggested a high level of ROS induced apoptosis and necroptosis simultaneously in GCs [30]. However, in certain cancer cell types, the increased level of ROS induces necroptosis selectively [31], and the induction of necroptosis was beneficial in apoptosis-resistant cancer cells [22].

Ferroptosis induction is an emerging strategy and existing anticancer agents such as CIS and CTX also may trigger ferroptotic pathways in cancer. In fact, ferroptosis could contribute more toxicity than apoptosis and necrosis in cancer [32], and these findings heighten interest in ferroptosis as a potential toxic mechanism for ovaries. It has been reported that the toxicity triggers follicle loss by increased ROS in both CTX- and CIS induced POI mice [6].While CIS-induced ferroptosis in GCs has been identified, it is unclear whether a relationship exists, and how the regulatory genes of ferroptosis are controlled in GCs in CTX-induced ovarian toxicity. In both animal models and patients, iron overload plays a pathogenic role in ovarian toxicity in several ovarian disorders, such as endometriosis-related infertility and polycystic ovary syndrome (PCOS) [33, 34]. Paradoxically, HO-1 is considered to have a cytoprotective role in ovarian function in several studies, as HO-1 deficiency causes deficient ovulation in mice [11, 12, 35]. On the other hand, HO-1-deficient mice developed an anemia associated with abnormally low serum iron levels [36]. Moreover, reports on the function of HO-1 are conflicting, with studies conversely describing it to promote or inhibit ferroptosis [37]. In models of cardiomyopathy and neurodegeneration, overexpression of HO-1 contributes to cell death [38, 39]. Consistent with these reports, our results confirmed cells with high HO-1 expression become cytotoxic due to sensitivity to ferroptosis.

GPX4 is considered one of the principal ferroptotic markers and plays a pivotal role in the onset of ferroptosis, as its inhibition triggers lipid perxidation, leading to cellular death. Indeed, HO-1 has been shown to be a regulator of iron metabolism and antioxidant capacity, and most ferroptosis inducers could inhibit GPX4 activity directly or indirectly [7]. Conversely, Zhang et al. showed that HO-1 and GPX4 were upregulated, it might suggest that HO-1 may mediate an enhanced anti-oxidative capacity in CIS-induced GC ferroptosis [6]. In contrast, CTX has been found to trigger ferroptosis by promoting HO-1 activity but had no effect on GPX4 expression in tumor cells [25]. However, our RNA-seq analysis and validated data confirmed that CTX-induced ferroptosis in GCs by upregulating the expression of HO-1, inhibiting the expression of GPX4, together with the depletion of GSH, and elevation of MDA.

Besides iron overload, lipid peroxidation is another fundamental element in ferroptosis. BNC mutation-induced POI mice exhibits iron overload and lipid peroxidation, evidenced by ferroptosis in oocytes [10]. Women with POI present abnormal lipid profile and dysregulation of fatty acid signals in GCs is a potential driver of human POI [40, 41]. In particular, lipid peroxides are in fact formed by ROS in the polyunsaturated acyl chains of membrane lipids. Oxidative stress can be induced by inhibition of GPX4 which promotes lipid deposition [42]. Furthermore, MDA was shown to accumulate in cells under oxidative stress, which is commonly used as an indicator of lipid peroxidation [26]. Our results suggest a decreased cellular anti-oxidant capacity, an increased lipid peroxidation and lipid accumulation to induce ferroptosis in response to CTX induction. Furthermore, Fer-1 and HO-1 konckdown could reverse the iron overload and increase in lipid peroxidation caused by CTX induction.

Although it is currently under debate whether mitochondria are involved in ferroptosis, the mechanism underlying POI appears to involve mitochondrial dysfunction in GCs [43]. We found that CTX caused an increase in mtROS, a loss of MMP and disruption of mitochondrial structure, while Fer-1 or HO-1 knockdown reversed these effects. It has been suggested that the altered level of HO-1 coordinates mitochondrial dysfunction [39]. Furthermore, GPX4 expression was shown to be induced in response to mitochondrial dysfunction in vitro and in vivo [44], supporting a role for mitochondria as an important site in ferroptosis regulation. Importantly, mitochondria are of utmost importance for a GC since they regulate redox homeostasis [18]. Besides, HO-1 is generally a cytoprotective mechanism in ferroptotic progression when HO-1 is moderately activated, however, a high degree of HO-1 activation could lead to ROS overload and subsequent cell death [25]. In this study, oxidative stress due to high cytoROS and mtROS, ROS levels was significantly increased in CTX-treated GCs tracked by DCFDA and MitoSOX™ and this could be moderated by ferroptosis inhibtion with Fer-1, indicating its potential application as a therapeutic agent against CTX toxicity. Our results may also underline the HO-1 overexpression and GPX depletion to induce high levels of ROS that might be facilitated by mitochondrial dysfunction in CTX-treated GCs. Inhibition of ferroptosis plays a positive role in CTX exposure through preserving mitochondrial function.

## Conclusion

In summary, CTX-induced ovarian toxicity was closely related to ferroptosis in GCs. The iron overload and disrupting ROS, including cytoROS, mtROS and lipROS homeostasis by HO-1 upregulation could induce ferroptosis via mitochondrial dysfunction in CTX-induced GCs. Moreover, HO-1 inhibition could suppress ferroptosis induced GPX4 depletion. This implies that there is a role for ROS in CTX-induced ferroptosis and highlighted the effect of HO-1 modulators in improving CTX-induced ovarian damage.

### Electronic supplementary material

Below is the link to the electronic supplementary material.

Additional file 1: figure S1 PPI network relationship graph of the ferroptosis-related genes (A) and OS-related genes (B).

Additional file 2: table S1 Primer sequences.

Additional file 3: table S2 Antibodies.

Additional file 4: table S3 Top 10 hub genes based on MCC score among co- expressed genes in DEGs and ferroptosis.

Additional file 5: table S4 Top 10 hub genes based on MCC score among co-expressed genes in DEGs and mitochondria.

## Data Availability

The data used during the current study are available from the corresponding author on reasonable request.
