# Supplementary figures and images for: Cyclophosphamide induces ovarian granulosa cell ferroptosis via a mechanism associated with HO-1 and ROS-mediated mitochondrial dysfunction
Source: J Ovarian Res. 2024 May 18;17:107. doi: 10.1186/s13048-024-01434-z (PMC11102268; doi:10.1186/s13048-024-01434-z)

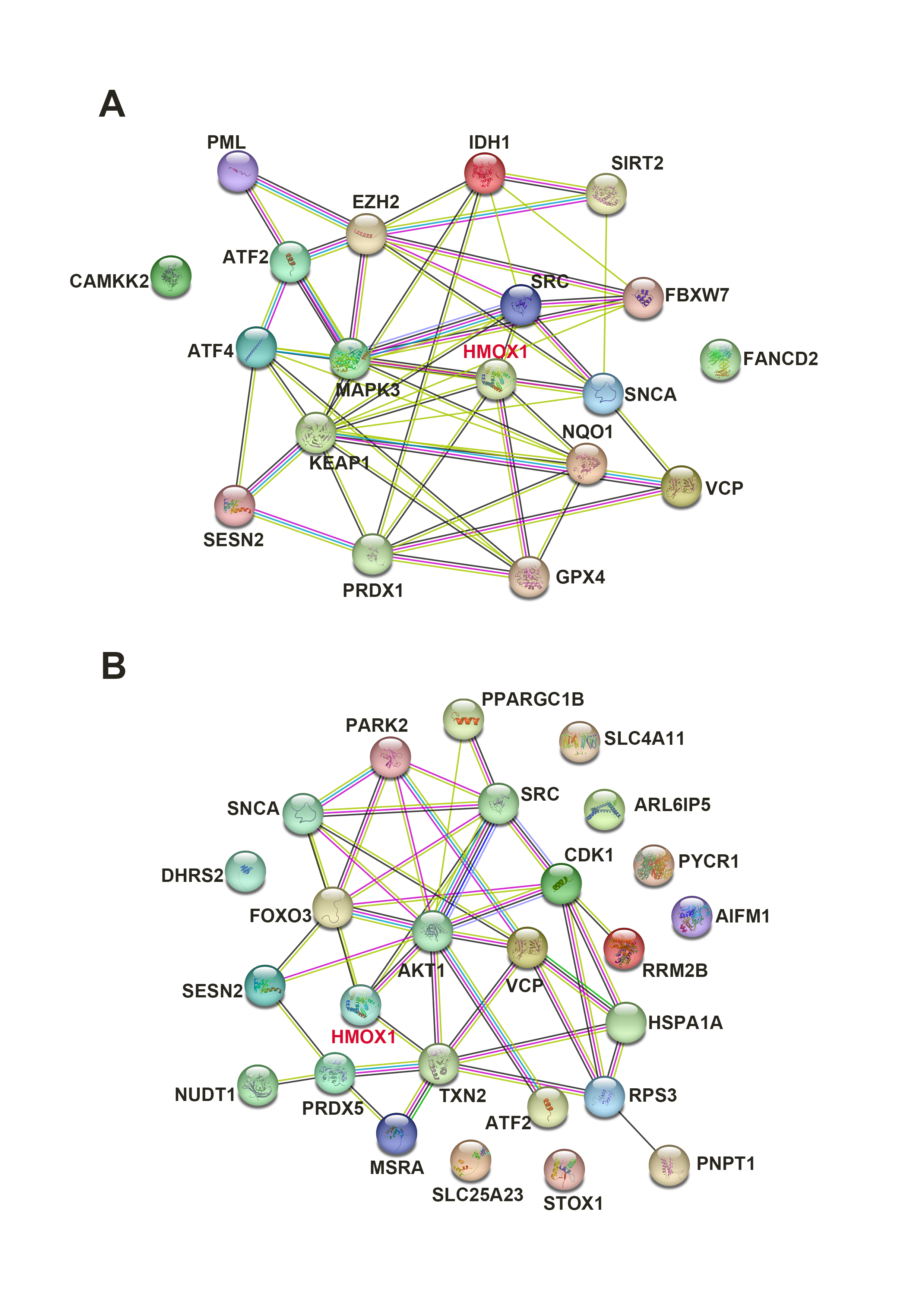

Supplement: Supplementary file 1 — Additional file 1: figure S1 PPI network relationship graph of the ferroptosis-related genes (A) and OS-related genes (B). [file 13048_2024_1434_MOESM1_ESM.jpg]
