# Supplementary material for: Cyclophosphamide induces ovarian granulosa cell ferroptosis via a mechanism associated with HO-1 and ROS-mediated mitochondrial dysfunction
Source: J Ovarian Res. 2024 May 18;17:107. doi: 10.1186/s13048-024-01434-z (PMC11102268; doi:10.1186/s13048-024-01434-z)
Supplement: Supplementary file 2 — Additional file 2: table S1 Primer sequences. [file 13048_2024_1434_MOESM2_ESM.docx]

**Table S1. Primer sequences.**

| **HUMAN-HO1** | Forward:5′-GCACCGGCCGGATGGAGCGTCC-3′;  Reverse:5′-CGTCTCGGGTCACCTGGCCCTTCTG-3′ |
| --- | --- |
| **HUMAN-GPX4** | Forward:5′-AGAGATCAAAGAGTTCGCCGC-3′;  Reverse:5′-TCTTCATCCACTTCCACAGCG-3′ |
| **HUMAN-GAPDH** | Forward:5′-GGAGCGAGATCCCTCCAAAAT-3′;  Reverse:5′-GGCTGTTGTCATACTTCTCATGG-3′ |
| **si-HO1_001** | 5′-CGTTCCTGCTCAACATCCA-3′ |
| **si- HO1_002** | 5′-CGATGGGTCCTTACACTCA-3′ |
| **si-HO1_003** | 5′-CAGTTGCTGTAGGGCTTTA-3′ |
