# Supplementary material for: Cyclophosphamide induces ovarian granulosa cell ferroptosis via a mechanism associated with HO-1 and ROS-mediated mitochondrial dysfunction
Source: J Ovarian Res. 2024 May 18;17:107. doi: 10.1186/s13048-024-01434-z (PMC11102268; doi:10.1186/s13048-024-01434-z)
Supplement: Supplementary file 3 — Additional file 3: table S2 Antibodies. [file 13048_2024_1434_MOESM3_ESM.docx]

**Table S2.List of Antibodies.**

| **Antibodies** | **Source** | **Identififier** |
| --- | --- | --- |
| Rabbit anti-HO-1 | Abcam (Cambridge, UK) | Cat# ab68477 |
| Rabbit anti-GPX 4 | Abcam (Cambridge, UK) | Cat# ab125066 |
| Mouse anti-β actin | Affifinity Biosciences (Cincinnati, OH) | Cat# T0022 |
