# Supplementary material for: Cyclophosphamide induces ovarian granulosa cell ferroptosis via a mechanism associated with HO-1 and ROS-mediated mitochondrial dysfunction
Source: J Ovarian Res. 2024 May 18;17:107. doi: 10.1186/s13048-024-01434-z (PMC11102268; doi:10.1186/s13048-024-01434-z)
Supplement: Supplementary file 4 — Additional file 4: table S3 Top 10 hub genes based on MCC score among co- expressed genes in DEGs and ferroptosis. [file 13048_2024_1434_MOESM4_ESM.docx]

**Table S3.** Top 10 hub genes based on MCC score among co-expressed genes in DEGs and ferroptosis.

| **Gene Symbol** | **Full Name** | **MCC Score** |
| --- | --- | --- |
| HO1 | homo sapiens heme oxygenase 1 | 38 |
| KEAP1 | kelch-like ECH-associated protein 1 | 34 |
| PRDX1 | peroxiredoxin 1 | 27 |
| NQO1 | NAD(P)H quinone dehydrogenase 1 | 26 |
| GPX4 | glutathione peroxidase 4 | 24 |
| MAPK3 | mitogen-activated protein kinase 3 | 20 |
| SRC | SRC proto-oncogene | 18 |
| SNCA | synuclein alpha | 15 |
| FBXW7 | f-box and WD repeat domain containing 7 | 13 |
| EZH2 | enhancer of zeste 2 polycomb repressive complex 2 subunit | 9 |
