# Supplementary material for: Cyclophosphamide induces ovarian granulosa cell ferroptosis via a mechanism associated with HO-1 and ROS-mediated mitochondrial dysfunction
Source: J Ovarian Res. 2024 May 18;17:107. doi: 10.1186/s13048-024-01434-z (PMC11102268; doi:10.1186/s13048-024-01434-z)
Supplement: Supplementary file 5 — Additional file 5: table S4 Top 10 hub genes based on MCC score among co-expressed genes in DEGs and mitochondria. [file 13048_2024_1434_MOESM5_ESM.docx]

**Table S4** Top 10 hub genes based on MCC score among co-expressed genes in DEGs and mitochondria.

| **Gene Symbol** | **Full Name** | **MCC Score** |
| --- | --- | --- |
| AKT1 | AKT serine/threonine kinase 1 | 68 |
| HSPA1A | heat shock protein family a member 1A | 51 |
| SNCA | synuclein alpha | 48 |
| SRC | SRC proto-oncogene | 36 |
| HO1 | heme oxygenase 1 | 30 |
| PARK2 | Parkinson disease 2 | 26 |
| VCP | valosin containing protein | 24 |
| FOXO3 | forkhead box O3 | 16 |
| CDK1 | cyclin dependent kinase 1 | 7 |
| PRDX5 | peroxiredoxin 5 | 4 |
